# Bacterial Load of *Gardnerella* spp. and *Fannyhessea vaginae* and Its Association with Cervicovaginal Inflammatory Cytokine Responses Across Vaginal Microbiota Patterns

**DOI:** 10.3390/microorganisms14030651

**Published:** 2026-03-13

**Authors:** Laura Emi Yonezawa, Jeniffer Sena Baptista Ferreira, Maria Eduarda Tesini Rocha, Rafael Gomes Barnabé, Hélio Amante Miot, Andréa da Rocha Tristão, Camila Marconi, Mariana de Castro Silva, Márcia Guimarães da Silva

**Affiliations:** 1 Department of Pathology, Botucatu Medical School, São Paulo State University (UNESP), Botucatu 18618-687, São Paulo, Brazil; laura.yonezawa@unesp.br (L.E.Y.); jeniffer.sena@unesp.br (J.S.B.F.); maria.tesini@unesp.br (M.E.T.R.); rafael.g.barnabe@unesp.br (R.G.B.); mariana.castro@unesp.br (M.d.C.S.); 2 Department of Dermatology, Botucatu Medical School, São Paulo State University (UNESP), Botucatu 18618-687, São Paulo, Brazil; helio.a.miot@unesp.br; 3 Department of Gynecology and Obstetrics, Botucatu Medical School, São Paulo State University (UNESP), Botucatu 18618-687, São Paulo, Brazil; andrea.tristao@unesp.br; 4 Department of Basic Pathology, Universidade Federal do Paraná (UFPR), Curitiba 81531-980, Paraná, Brazil; marconi.cml@gmail.com

**Keywords:** *Gardnerella* spp., *Fannyhessea vaginae*, vaginal microbiota, bacterial vaginosis, inflammatory cytokines

## Abstract

Bacterial vaginosis (BV) is a common vaginal dysbiosis characterized by the depletion of *Lactobacillus* species and the overgrowth of facultative anaerobic bacteria, particularly *Gardnerella* spp. and *Fannyhessea vaginae*. The vaginal microbiota plays a key role in local immune modulation, and BV has been associated with a molecular pro-inflammatory profile. This study included 152 women with normal microbiota (n = 68), intermediate microbiota (n = 24), or BV (n = 60). Vaginal lavage samples were used to quantify *Gardnerella* spp. and *F. vaginae* and to measure IL-1β, IL-6, CXCL-8, IL-10, and TNF-α levels. Bacterial loads of *Gardnerella* spp. were significantly higher in the BV group than in normal microbiota (*p* < 0.001). *F. vaginae* loads were higher in BV than in both normal and intermediate microbiota (*p* < 0.001). IL-1β levels were increased in intermediate microbiota (*p* = 0.011) and BV (*p* = 0.024) compared with normal microbiota, while CXCL-8 levels were higher in intermediate microbiota (*p* = 0.021). No differences were observed for IL-6, IL-10, or TNF-α. BV is associated with increased *Gardnerella* spp. and *F. vaginae* loads and a selective increase in IL-1β, supporting a distinct inflammatory signature linked to vaginal dysbiosis.

## 1. Introduction

Human vaginal microbiota establish a mutualistic relationship with the host, playing a critical role in immune modulation and protection against pathogenic microorganisms. A healthy vaginal microenvironment is typically dominated by *Lactobacillus* species, which produce lactic acid, hydrogen peroxide, and bacteriocins, contributing to the maintenance of an acidic vaginal pH (3.8–4.5) and inhibiting the growth of bacterial and viral pathogens, including *Chlamydia trachomatis*, *Neisseria gonorrhoeae*, *Mycoplasma genitalium*, human papillomavirus (HPV), and human immunodeficiency virus (HIV) [1,2]. The maintenance of a eubiotic vaginal microbiota has also been consistently associated with favorable reproductive outcomes, including improved success rates in assisted reproductive technologies and reduced risk of adverse pregnancy outcomes [3,4].

As a dynamic ecosystem, vaginal microbiota is influenced by hormonal fluctuations, pregnancy, menstrual cycle, and external factors such as contraceptive use, stress, and sexual and hygiene practices [5,6]. Disruption of *Lactobacillus*-dominated communities and overgrowth of anaerobic bacteria lead to vaginal dysbiosis, most notably bacterial vaginosis (BV). BV is characterized by a marked reduction or absence of *Lactobacillus* species and increased abundance of facultative anaerobic bacteria, including *Gardnerella vaginalis*, *Fannyhessea vaginae*, *Mycoplasma hominis*, *Ureaplasma urealyticum*, *Prevotella bivia*, *Mobiluncus* spp., and *Leptotrichia* spp. [7,8]. BV prevalence ranges from 5% to 70% worldwide, varies according to population characteristics and diagnostic criteria, and is frequently asymptomatic, contributing to underdiagnosis and persistence [9,10,11,12].

Among BV-associated bacteria, *G. vaginalis* has historically been considered a key species in BV pathogenesis due to its ability to colonize the vaginal epithelium, evade host immune responses, and form structured biofilms through virulence factors such as sialidase A and vaginolysin [13,14,15,16,17]. *F. vaginae*, formerly *Atopobium vaginae*, is also frequently detected within the pathogenic core of BV and has been proposed as an indicator species, being present in up to 95% of BV cases, most often in co-occurrence with *G. vaginalis* [18,19,20,21]. This synergistic interaction is thought to contribute to biofilm stability and BV persistence.

Vaginal microbiota plays a central role in mucosal immune regulation. Although BV has traditionally been considered a non-inflammatory condition, it is increasingly recognized as a state of molecular inflammation characterized by altered cytokine and chemokine profiles. Anaerobe-dominated communities are associated with enhanced pro-inflammatory responses, whereas *Lactobacillus*-dominated microbiota promote antimicrobial defense without excessive inflammation. Notably, *G. vaginalis* and *F. vaginae* appear to exert distinct, species-specific effects on host immune responses, potentially influencing BV persistence and its association with sexually transmitted infections and adverse reproductive outcomes [22,23,24]. Therefore, this study aimed to quantify *Gardnerella* spp. and *F. vaginae* across different vaginal microbiota patterns and to evaluate their association with cervicovaginal cytokine responses in women of reproductive age.

## 2. Materials and Methods

### 2.1. Study Design and Population

This prospective, cross-sectional study included 152 women attending the Female Genital Infections Outpatient Clinic at Botucatu Medical School University Hospital Complex (HCFMB–UNESP), Brazil, between 2024 and 2025. At enrollment, participants completed a structured questionnaire to collect sociodemographic information, sexual and behavioral habits, and gynecological history. All participants provided written informed consent prior to inclusion in the study. The study protocol was approved by the local Research Ethics Committee (CAAE: 91235025.0.0000.5411).

Eligible participants were women aged 18 years or older who were of reproductive age, non-pregnant, had not received treatment for genital tract infections within the preceding 30 days, reported sexual abstinence for at least 48 h prior to sample collection, and were at least five days beyond the end of menstruation. Three distinct biological samples were collected from each participant. First, a vaginal swab was obtained from the middle third of the lateral vaginal wall and used for microscopic evaluation of the vaginal microbiota, including Gram staining and microbiota classification. Second, an endocervical sample was collected using a cytobrush and used exclusively for molecular screening of sexually transmitted infections (*N. gonorrhoeae*, *C. trachomatis*, *Trichomonas vaginalis*, and *M. genitalium*). Third, a cervicovaginal lavage sample was obtained by instilling 3 mL of sterile 0.9% saline solution onto the vaginal walls, gently mixing with cervicovaginal secretions, and recovering the fluid using a sterile plastic pipette. This lavage sample was used for the quantification of *Gardnerella* spp. and *F. vaginae* by quantitative PCR and for ELISA to assess cytokine levels.

Exclusion criteria included immunosuppression, history of solid organ transplantation, seropositivity for HIV or syphilis, and a positive endocervical molecular test for *C. trachomatis*, *N. gonorrhoeae*, *T. vaginalis*, or *M. genitalium*.

### 2.2. Microscopic Diagnosis and Vaginal Sample Collection

At study enrollment, a sterile, lubricant-free bivalve vaginal speculum was used for examination. Vaginal samples were collected using a sterile swab from the middle third of the lateral vaginal wall and used to prepare Gram-stained smears for microscopic evaluation of the vaginal microbiota. Microscopic classification of the vaginal microbiota was performed according to the Nugent scoring system, a standardized and widely accepted method based on the semi-quantitative assessment of bacterial morphotypes observed on Gram-stained smears. Specifically, three bacterial morphotypes are evaluated: (i) large Gram-positive rods, predominantly *Lactobacillus* species; (ii) small Gram-variable rods, consistent with *Gardnerella* and *Bacteroides* morphotypes; and (iii) curved Gram-variable rods, consistent with *Mobiluncus* species. Each morphotype is assigned a score ranging from 0 to 4 according to its relative abundance, and the total Nugent score is calculated by summing the individual scores. According to the original criteria described by Nugent et al., total scores of 0–3, 4–6, and 7–10 correspond to normal microbiota, intermediate microbiota, and bacterial vaginosis (BV), respectively. Throughout the manuscript, vaginal microbiota status refers to ecological states inferred from Nugent morphotype scoring of Gram-stained vaginal smears.

Additional microscopic criteria described in the literature were applied exclusively to identify conditions in which Nugent scoring is not applicable, such as cytolytic vaginosis (Cibley and Cibley, 1991) [25] and aerobic vaginitis (Dong et al., 2022) [26]. Participants diagnosed with these conditions were excluded from the study and were referred for clinical management in accordance with applicable clinical guidelines. Based on Nugent score classification, participants included in the analysis were categorized as having normal microbiota (n = 68), intermediate microbiota (n = 24), or bacterial vaginosis (n = 60).

Endocervical samples were collected for molecular screening of *N. gonorrhoeae*, *C. trachomatis*, *T. vaginalis*, and *M. genitalium* by real-time PCR. Cervical specimens were placed in 15 mL Falcon tubes containing 1000 µL of Tris–HCl buffer (50 mM, pH 8.5) supplemented with EDTA (1 mM, pH 8.0) and stored at −80 °C until processing. Vaginal lavage samples were also obtained for the detection and quantification of *Gardnerella* spp. and *F. vaginae*, as well as for cytokine measurements. Briefly, 3 mL of sterile 0.9% saline solution was instilled onto the vaginal wall, gently mixed with cervicovaginal secretions, and recovered using a sterile plastic pipette. Lavage samples were stored at −80 °C until further analysis.

### 2.3. Molecular Screening for Sexually Transmitted Infections

DNA was extracted from endocervical samples using the QIAamp DNA Mini Kit (Qiagen, Hilden, Germany), according to the manufacturer’s instructions. DNA integrity and amplifiability were verified by amplification of the constitutive β-globin gene using the PCO4 and GH20 primers, as previously described [27]. Molecular screening for *N. gonorrhoeae*, *C. trachomatis*, *T. vaginalis*, and *M. genitalium* was performed using a multiplex real-time PCR assay (Allplex™ CT/NG/MG/TV Assay; Seegene, Seoul, Republic of Korea), which employs multiple targets and pathogen-specific probes for simultaneous detection of the four microorganisms. PCR reactions were carried out on a CFX96™ Real-Time PCR Detection System (Bio-Rad, Hercules, CA, USA) in accordance with the manufacturer’s recommendations.

Each run included an internal control to monitor PCR inhibition, as well as positive and negative controls. Target detection was based on automatically generated cycle threshold (Ct) values, and result interpretation (positive, negative, or invalid) was performed using Seegene Viewer software (V1.00.001), following the criteria established by the manufacturer.

### 2.4. Quantification of Gardnerella spp. by Quantitative PCR

DNA was extracted from cervicovaginal lavage samples using the DNeasy PowerSoil Pro Kit (Qiagen, Hilden, Germany), according to the manufacturer’s recommendations. To generate a standard curve, *G. vaginalis* (ATCC^®^ 14018™) obtained from the American Type Culture Collection (ATCC) was cultured in New York City III (NYC III) medium containing 0.4% HEPES, 1.5% peptone, 0.5% sodium chloride, 0.4% resazurin, 0.5% glucose, 0.375% yeast extract, 10% horse serum, and 85.1% deionized water, with 1.5% agar added when required. Cultures were incubated under anaerobic conditions using an anaerobiosis generator (Anaerobac; Probac, #ANA16T, São Paulo, Brazil) for 24–48 h.

For cloning, bacterial cultures were used to amplify a 332 bp fragment of the 16S rRNA gene by conventional PCR using the primers F-GV1 (5′-TTACTGGTGTATCACTGTAAGG-3′) and R-GV3 (5′-CCGTCACAGGCTGAACAGT-3′) [28]. Following confirmation of the expected amplicon, PCR products were purified using the Illustra GFX PCR DNA and Gel Band Purification Kit (Cytiva, Bangalore, India) and ligated into the pJET1.2/blunt cloning vector using T4 DNA ligase, according to the manufacturer’s instructions. The ligation mixture was cloned into *Escherichia coli* DH5-α cells (Invitrogen, Carlsbad, CA, USA) by electroporation and heat shock. Transformed cells were cultured on Luria–Bertani (LB) medium supplemented with ampicillin as a selective antibiotic. Plasmid DNA was extracted using the GeneJET Plasmid Miniprep Kit (Thermo Fisher Scientific, Vilnius, Lithuania), according to the manufacturer’s instructions. Recombinant colonies were screened by PCR using internal primers specific to the cloned fragment. Plasmid DNA concentration was determined using the Qubit™ dsDNA Broad Range (BR) Assay, and the insert sequence was validated by Sanger sequencing on a 500 Genetic Analyzer (Applied Biosystems, Foster City, CA, USA) and confirmed by BLAST+ 2.17.0 analysis.

Absolute quantification of *Gardnerella* spp. was performed by quantitative real-time PCR (qPCR) using 2× qPCRBIO SyGreen Hi-ROX Master Mix (PCR Biosystems, London, UK) and the primers F-GV1 and R-GV3. Reactions were carried out in a final volume of 20 μL, containing 4 μL of extracted DNA (20 ng/μL), under the following cycling conditions: initial denaturation at 95 °C for 2 min, followed by 40 cycles of denaturation at 95 °C for 5 s, annealing at 60 °C for 25 s, and extension at 72 °C for 15 s. A melting curve analysis was subsequently performed (95 °C for 15 s, 60 °C for 1 min, and 95 °C for 15 s) using a StepOnePlus™ Real-Time PCR System (Thermo Fisher Scientific, Waltham, MA, USA). Samples presenting a melting temperature of 87 ± 1 °C were considered positive. Standard curves were generated using three plasmid dilutions (10^5^, 10^6^, and 10^7^ copies), allowing the establishment of a linear relationship between cycle threshold (Ct) values and *Gardnerella* spp. copy number. All reactions were performed in triplicate.

### 2.5. Quantification of Fannyhessea vaginae by Quantitative PCR

For construction of the standard curve, a synthetic plasmid containing a 301 bp fragment of the 16S rRNA gene of *F. vaginae* was designed and synthesized (GENEART, Thermo Fisher Scientific). The plasmid was linearized using NotI FastDigest (Thermo Fisher Scientific), quantified using the Qubit™ dsDNA High Sensitivity (HS) Assay, and purified with the Illustra GFX PCR DNA and Gel Band Purification Kit (Cytiva, Bangalore, India).

Absolute quantification of *F. vaginae* was performed by quantitative real-time PCR (qPCR) using 2× qPCRBIO SyGreen Hi-ROX Master Mix (PCR Biosystems, London, UK) and the primers Fw (5′-TAGGCGGTYTGTTAGGTCAGGA-3′) and Rv (5′-CCTACCAGACTCAAGCCTGC-3′) [28]. Reactions were carried out in a final volume of 20 μL, containing 4 μL of extracted DNA (20 ng/μL), under the following cycling conditions: initial denaturation at 95 °C for 2 min, followed by 40 cycles of denaturation at 95 °C for 5 s, annealing at 60 °C for 25 s, and extension at 72 °C for 15 s. A melting curve analysis was then performed (95 °C for 15 s, 60 °C for 1 min, and 95 °C for 15 s) using a StepOnePlus™ Real-Time PCR System (Thermo Fisher Scientific, Waltham, MA, USA). Samples presenting a melting temperature of 83.2 ± 0.5 °C were considered positive for *F. vaginae*. Standard curves were generated using five serial plasmid dilutions (5 × 10^8^, 5 × 10^7^, 5 × 10^6^, 5 × 10^5^, and 5 × 10^4^ copies/μL), enabling the establishment of a linear relationship between Ct values and *F. vaginae* copy number. All samples were analyzed in triplicate.

### 2.6. Cytokine Quantification

To assess inflammatory cytokine production, cervicovaginal lavage samples collected from the middle third of the vaginal wall were used for the quantification of IL-1β, IL-6, CXCL-8, IL-10, and TNF-α. These cytokines were selected based on their well-established roles in vaginal innate immune responses and bacterial vaginosis-associated inflammation, encompassing key pro-inflammatory mediators involved in epithelial activation and immune cell recruitment (IL-1β, IL-6, CXCL-8, and TNF-α), as well as the anti-inflammatory/regulatory cytokine IL-10, which contributes to immune homeostasis in the vaginal microenvironment.

Prior to analysis, samples were centrifuged to remove cellular debris, and supernatants were aliquoted and stored at −80 °C until use. Cytokine concentrations were measured using commercially available DuoSet ELISA kits (R&D Systems, Minneapolis, MN, USA), following the manufacturer’s protocols. Briefly, 96-well microplates were coated overnight at room temperature with capture antibodies diluted in PBS, washed, and blocked for 1 h. Subsequently, 100 μL of samples or standards was added to each well and incubated for 4 h at room temperature. After washing, detection antibodies were added and incubated for 2 h, followed by incubation with streptavidin–HRP for 20 min. Color development was achieved using substrate solution, and the reaction was stopped after 20 min. Absorbance values were measured at 492 nm using an automated ELISA plate reader (BioTek Instruments Inc., Winooski, VT, USA). The limits of detection for IL-1β, IL-6, CXCL-8, IL-10, and TNF-α were 3.9 pg/mL, 9.4 pg/mL, 15.6 pg/mL, 15.6 pg/mL, and 15.6 pg/mL, respectively.

### 2.7. Statistical Analysis

Statistical analyses were performed using R software (version 2025.09.0-387). Bacterial loads of *Gardnerella* spp., *F. vaginae*, and the combined bacterial burden (*Gardnerella* spp. + *F. vaginae*) were log-transformed due to data skewness and differences in orders of magnitude across measurements. For descriptive analyses of vaginal microbiota groups (normal microbiota, intermediate microbiota and BV), median values and interquartile ranges (25th–75th percentiles) were calculated. Zero values were excluded from log-transformed analyses, as logarithmic transformation is not applicable to zero. Comparisons among microbiota groups were performed using the Kruskal–Wallis test, followed by Dunn’s post hoc test with Bonferroni correction. A significance level of *p* < 0.05 was adopted.

Correlations between cytokine levels (IL-1β, IL-6, CXCL-8, IL-10, and TNF-α) and bacterial variables (*Gardnerella* spp., *F. vaginae*, and *Gardnerella* spp. + *F. vaginae*) were assessed using Spearman’s rank correlation coefficient (ρ), given the non-parametric distribution of the data. Scatter plots were generated to illustrate the relationships between bacterial load and cervicovaginal cytokine levels, with each point representing an individual sample. Bacterial loads are expressed as log_10_-transformed DNA copy numbers, while cytokine concentrations are also presented on a log_10_ scale to account for non-normal data distribution. Samples are color-coded according to vaginal microbiota classification (normal microbiota, intermediate microbiota, and bacterial vaginosis), allowing visualization of microbiota-specific patterns.

Associations between bacterial variables and cytokine levels were evaluated using Spearman’s rank correlation coefficient, which was selected due to the non-parametric distribution of both bacterial load and cytokine data. Correlation strength was interpreted based on Spearman’s ρ values, and statistical significance was defined as *p* < 0.05.

## 3. Results

### 3.1. Sociodemographic Characteristics

The sociodemographic characteristics of the 152 study participants are presented in Table 1. The median ages were 37 years (22–54) in women with normal microbiota, 39 years (19–55) withintermediate microbiota, and 41 years (20–58) with bacterial vaginosis. Most participants self-identified as white, regardless of the microbiota profile. Among intermediate microbiota cases, married women predominated, whereas a higher proportion of single women was observed in the BV group. Most patients did not report vaginal discharge, odor, or pruritus, irrespective of the microscopic diagnosis of normal, intermediate microbiota or BV. A subset of participants did not provide information on symptoms, corresponding to 24.3% for vaginal discharge, 40.8% for odor, and 33.5% for pruritus.

### 3.2. Quantitative Profile of Bacterial Species Associated with BV

**Figure 1:**
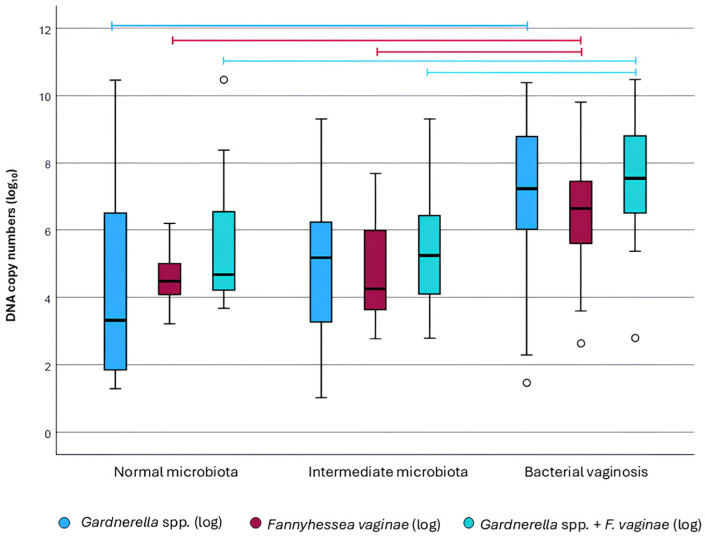
Bacterial load of *Gardnerella* spp., *Fannyhessea vaginae*, and their combined abundance across normal microbiota, intermediate microbiota, and bacterial vaginosis (BV) groups. Bacterial loads were quantified by absolute qPCR and are expressed as DNA copy numbers (log_10_). Boxplots represent the median and interquartile range. Individual data points falling outside the whisker range are shown as small white circles and represent statistical outliers. Comparisons between microbiota groups were performed using the Kruskal–Wallis test followed by Dunn’s multiple comparisons test. Horizontal bars indicate statistically significant differences between groups (*p* < 0.05). Blue bars indicate statistical differences in *Gardnerella* spp. load, burgundy bars indicate differences in *F. vaginae* load, and turquoise bars represent combined *Gardnerella* spp. + *F. vaginae* bacterial load.

Among the 152 participants included in the study, 95 (62.5%) tested positive for *Gardnerella* spp. Of these, 35 were classified as normal microbiota (36.8%), 14 as intermediate microbiota (14.7%), and 46 as BV (48.4%). In normal microbiota cases, the median *Gardnerella* spp. bacterial load was approximately 3.4 copies/µL (1.8–6.7), whereas in BV cases, the median was significantly higher at 7.2 copies/µL (6.1–8.7) (*p* < 0.001). In intermediate microbiota, the median load was 6.2 copies/µL (3.4–8.6), with no statistically significant difference compared to normal microbiota [3.4 copies/µL (1.8–6.7)] or BV [7.2 copies/µL (6.1–8.7)] (*p* > 0.05) (Figure 1).

Regarding *F. vaginae*, the species was detected in 106 (69.7%) vaginal samples. Of these, 51 (48.1%) belonged to normal microbiota, 15 (14.2%) to intermediate microbiota, and 40 (37.7%) to BV. Considering the vaginal microbiota profiles, 75.0% (51/68) of normal microbiota cases were positive for *F. vaginae*, compared with 63.0% (15/24) of intermediate microbiota cases and 66.7% (40/60) of BV cases. In normal microbiota, the median bacterial load was 4.4 copies/µL (3.9–5.1), which was significantly lower than in BV [6.5 copies/µL (5.5–7.5); *p* < 0.001]. Intermediate microbiota cases showed a median of 4.5 copies/µL (3.7–5.4), with no significant difference compared to eubiosis, but with significantly lower values than those observed in BV (*p* < 0.001) (Figure 1).

Considering the synergistic interaction between *Gardnerella* spp. and *F. vaginae* in the formation of the characteristic BV biofilm, the combined bacterial loads of both species were also evaluated. A total of 74 (48.7%) women showed simultaneous positivity, of whom 28 (37.8%) were classified as normal microbiota, 10 (13.5%) as intermediate microbiota, and 36 (48.7%) as BV. The median combined loads were 4.6 copies/µL (4.2–6.5) for normal microbiota, 5.2 copies/µL (4.1–6.4) for intermediate microbiota, and 7.5 copies/µL (6.5–8.8) for BV. The combined bacterial loads followed the same trend observed for *F. vaginae* alone, with significantly higher values associated with dysbiosis compared to eubiosis (*p* < 0.001) and intermediate microbiota (*p* = 0.006). No statistically significant difference was observed between normal and intermediate microbiota (Figure 1).

### 3.3. Inflammatory Cytokines Associated with the Vaginal Microbiota Profile

**Figure 2:**
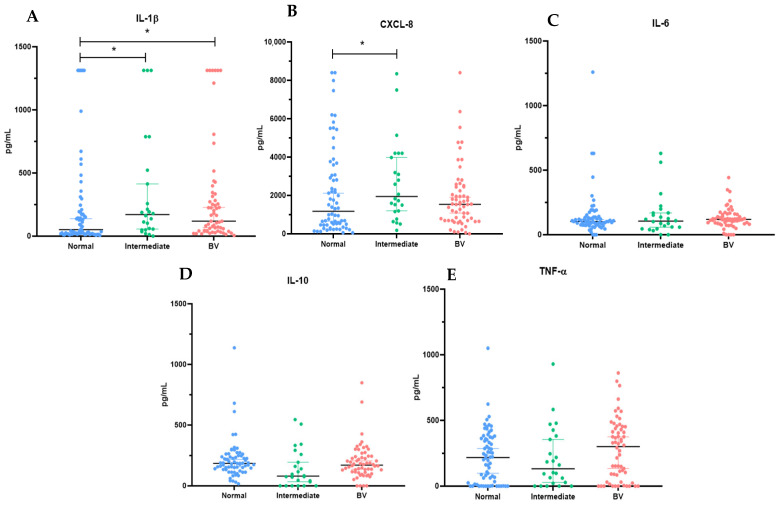
Quantitative representation of cytokine levels in cervicovaginal lavages from women according to the vaginal microbiota profile classified as normal microbiota, intermediate microbiota, and bacterial vaginosis (BV). Concentrations of (**A**) IL-1β, (**B**) CXCL-8, (**C**) IL-6, (**D**) IL-10, and (**E**) TNF-α (pg/mL). Bars represent the median with dispersion of individual absolute values. * indicates statistical significance.

To investigate whether variations in bacterial load are associated with modulation of the local immune response, we quantified a panel of cytokines representative of key inflammatory pathways in the vaginal microenvironment, including pro-inflammatory mediators involved in epithelial activation and immune cell recruitment (IL-1β, IL-6, CXCL-8, and TNF-α), as well as the regulatory cytokine IL-10, which contributes to immune homeostasis. IL-1β levels were significantly higher in samples from patients diagnosed with intermediate microbiota [2.3 pg/mL (1.8–2.7); *p* = 0.011] and BV [2.1 pg/mL (1.6–2.5); *p* = 0.024] compared with women classified as normal microbiota [1.7 pg/mL (1.3–2.3)]. No statistically significant differences were observed between the intermediate microbiota [2.3 pg/mL (1.8–2.7)] and BV [2.1 pg/mL (1.6–2.5)] groups (*p* = 0.408) (Figure 2A).

CXCL-8 levels also differed significantly, being higher in the intermediate microbiota group [3.3 pg/mL (3.1–3.6)] compared with the normal microbiota group [3.1 pg/mL (2.7–3.5)] (*p* = 0.021). However, no significant differences were observed between normal microbiota [3.1 pg/mL (2.7–3.5)] and BV [3.2 pg/mL (2.8–3.4)] (*p* = 0.442), nor between intermediate microbiota [3.3 pg/mL (3.1–3.6)] and BV [3.2 pg/mL (2.8–3.4)] (*p* = 0.075) (Figure 2B). The other cytokines analyzed—IL-6, IL-10, and TNF-α—did not show significant differences among the vaginal microbiota patterns defined according to the criteria of Nugent [11] (Figure 2C–E).

**Figure 3:**
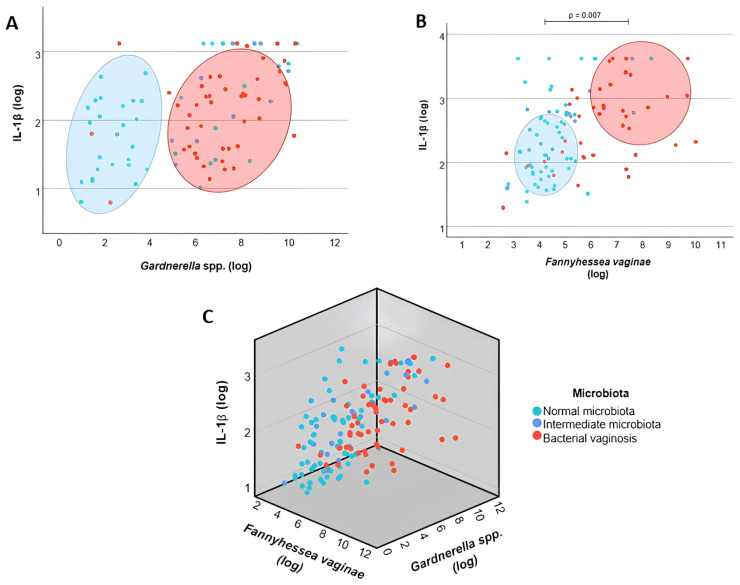
Two-dimensional (2D) and three-dimensional (3D) scatter plot representations showing the relationship between bacterial loads and IL-1β levels in cervicovaginal lavages. (**A**) Association between *Gardnerella* spp. bacterial load and IL-1β levels. (**B**) Association between *Fannyhessea vaginae* bacterial load and IL-1β levels. (**C**) Three-dimensional scatter plot illustrating the combined relationship between *Gardnerella* spp. and *F. vaginae* loads and IL-1β levels. Bacterial loads and cytokine concentrations are expressed as log_10_-transformed values. Green dots represent women with normal microbiota, blue dots represent intermediate microbiota, and red dots represent bacterial vaginosis.

**Figure 4:**
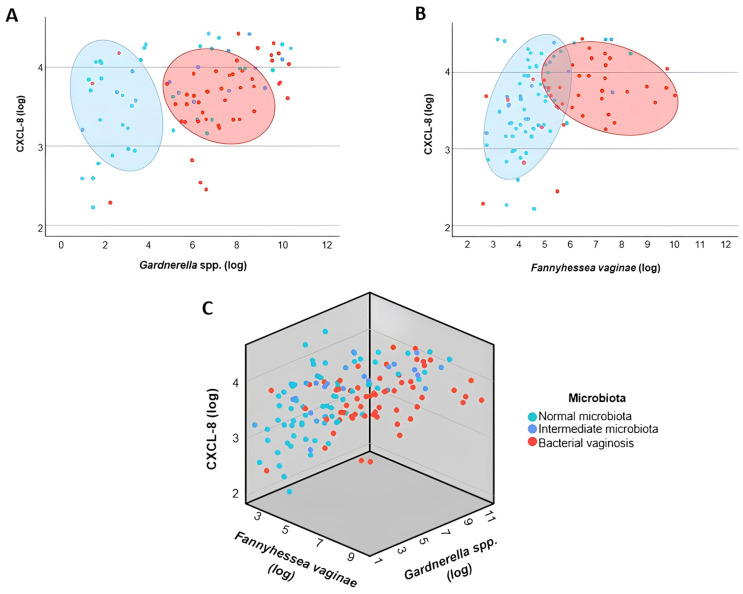
Two-dimensional (2D) and three-dimensional (3D) scatter plot representations showing the relationship between bacterial loads and CXCL-8 levels in cervicovaginal lavages. (**A**) Association between *Gardnerella* spp. bacterial load and CXCL-8 levels. (**B**) Association between *Fannyhessea vaginae* bacterial load and CXCL-8 levels. (**C**) Three-dimensional scatter plot illustrating the combined relationship between *Gardnerella* spp. and *F. vaginae* loads and CXCL-8 levels. Bacterial loads and cytokine concentrations are expressed as log_10_-transformed values. Green dots represent women with normal microbiota, blue dots represent intermediate microbiota, and red dots represent bacterial vaginosis.

After the individual evaluation of bacterial and immunological variables, multivariate analyses were performed to investigate the association between *Gardnerella* spp. and *F. vaginae* loads and inflammatory cytokine levels, while simultaneously considering the clinical classification of the vaginal microbiota (normal, intermediate and BV). In the adjusted model, both *F. vaginae* load (*p* = 0.007) and clinical microbiota classification (*p* = 0.001) remained associated with IL-1β levels, indicating that progressively more dysbiotic profiles exhibited higher *F. vaginae* loads and increased levels of this cytokine. In contrast, *Gardnerella* spp. load lost statistical significance in the adjusted model (*p* = 0.079) (Figure 3A–C). These findings suggest that the progression of vaginal dysbiosis—from normal microbiota to intermediate microbiota and to bacterial vaginosis—is associated with a concomitant increase in *F. vaginae* load and in the molecular inflammatory response, as reflected by higher IL-1β levels. Regarding CXCL-8, only the clinical microbiota classification remained associated with the levels of this chemokine (*p* = 0.004), whereas *Gardnerella* spp. and *F. vaginae* loads did not show independent associations (*p* = 0.058 and *p* = 0.516, respectively) (Figure 4A–C).

## 4. Discussion

Bacterial vaginosis (BV), the main dysbiotic condition of the female lower genital tract, is characterized not only by a reduction in *Lactobacillus* spp. but also by an increase in anaerobic bacterial species, particularly *G. vaginalis* and *F. vaginae*. Although the presence of these species in BV is well documented, gaps remain regarding the impact of their bacterial loads and their relationship with modulation of the inflammatory response in the vaginal microenvironment. The present study aimed to address this gap by evaluating the quantitative profiles of *Gardnerella* spp. and *F. vaginae* in women with different vaginal microbiota patterns, as well as their association with inflammatory mediators.

Our main findings reaffirm that both species are present across different vaginal microbiota profiles, indicating that their presence alone does not define a dysbiotic state, but rather their abundance. In this context, bacterial loads—particularly of *F. vaginae*—were significantly higher in the BV group, as was the combined load of both species, suggesting that the quantitative increase in these microorganisms is associated with dysbiosis progression. From an inflammatory perspective, a selective increase in IL-1β was observed in the intermediate microbiota and BV groups, and *F. vaginae* load remained associated with this elevation.

The detection of *Gardnerella* spp. in *Lactobacillus*-dominated microbiota is not unexpected and has been widely reported in the literature, reinforcing that the presence of this species alone is insufficient to characterize vaginal dysbiosis [29,30]. Microbiota classification-based studies, such as the VALENCIA model, have demonstrated that *Gardnerella* spp. can be detected across different CSTs, with varying abundance even in profiles considered eubiotic [31]. In that study, *Gardnerella* spp. were present at moderate abundance in CST IV-A and at high abundance in CST IV-B. In the present study, although *Gardnerella* spp. was detected across all vaginal microbiota profiles, its absolute bacterial load was significantly higher in BV compared with normal microbiota, supporting its role as a central component of dysbiosis. This finding is consistent with previous quantitative studies reporting elevated *Gardnerella* spp. loads in BV and low bacterial burdens in *Lactobacillus*-dominated microbiota [32,33,34]. In contrast, data regarding absolute *Gardnerella* spp. load in intermediate microbiota remain limited in the literature, as most studies focus primarily on binary comparisons between normal microbiota and BV.

However, no statistically significant difference was observed between BV and intermediate microbiota. This finding may reflect the heterogeneous nature of intermediate microbiota, which is often considered a transitional state between eubiosis and dysbiosis. A systematic review analyzing molecular characterization studies of the vaginal microbiota reported similar results, showing no significant difference in *G. vaginalis* bacterial load between intermediate microbiota and BV in most studies [34]. Another possible explanation for this finding relates to a limitation of the present study, namely the smaller sample size of the intermediate microbiota group in our cohort, as the number of intermediate microbiota cases was less than half that of normal microbiota and BV cases.

Regarding *F. vaginae*, our results showed that its bacterial load was similar between normal and intermediate microbiota, but significantly different from both when compared with BV. The higher load observed in BV suggests that this species is more strongly associated with established vaginal dysbiosis than with intermediate stages of microbial transition. In the literature, findings regarding the presence of *F. vaginae* in women without BV are heterogeneous [20,35,36]. While some studies report low prevalence of this species in *Lactobacillus*-dominated microbiota [35,36], others have identified it at higher proportions [20]. This variability has been attributed to methodological differences, population characteristics, and analytical sensitivity of the tests employed. Nevertheless, the consistent increase in its load in BV cases reinforces its role as an important microbial marker of vaginal dysbiosis.

Our results are also consistent with previous studies showing substantially higher loads of *Gardnerella* spp. in association with *F. vaginae* in dysbiotic microbiota, as well as demonstrating that the coexistence of these species at high concentrations increases the predictive value for BV diagnosis [17,29,31,36,37,38]. Santos-Greatti et al. [39] and Marconi et al. [28] reported similar findings, observing higher bacterial loads in women with BV and reinforcing the role of these species as central components of the polymicrobial core associated with dysbiosis.

Importantly, unlike most studies available in the literature, the present work included the evaluation of intermediate microbiota, which is frequently excluded from analyses. Our findings indicate that *Gardnerella* spp. does not reach significantly elevated levels during transitional stages of the microbiota, whereas *F. vaginae* appears to be more closely associated with established dysbiosis, although its load begins to increase already in intermediate microbiota. This pattern suggests that intermediate microbiota presents intermediate bacterial loads of core BV-associated species, reinforcing the hypothesis that it represents a transitional state between eubiosis and dysbiosis and highlighting the need for greater attention to this profile in future studies.

In addition, the composition of the vaginal microbiota plays a decisive role in maintaining ecological stability and the physiological balance of the vaginal microenvironment [1,2]. This ecosystem directly influences epithelial barrier integrity and the modulation of the local immune response. Disruptions in this balance may favor the proliferation of dysbiosis-associated species, including those of interest in the present study. Accumulating evidence indicates that *G. vaginalis* and *F. vaginae* contribute to the organization of structured biofilms and to the modulation of the vaginal immune response, playing a central role in the establishment and persistence of BV [7,8].

*G. vaginalis* exhibits a range of virulence factors that promote colonization, immune evasion, and biofilm formation, notably sialidase A—which degrades components of the vaginal epithelial barrier—and vaginolysin, a cholesterol-dependent cytolysin responsible for pore formation in epithelial cells, facilitating cell lysis [14,22]. These mechanisms compromise epithelial integrity and create a microenvironment favorable to bacterial adhesion and colonization by other BV-associated species. *F. vaginae*, in turn, typically occurs in association with *G. vaginalis* and shows a high capacity for biofilm integration, contributing to its structural stability and resistance to antimicrobial treatment [21]. The synergistic interaction between these species promotes the persistence of dysbiosis and is associated with the maintenance of a non-quiescent environment characteristic of BV. Thus, bacterial virulence factors, combined with biofilm organization, enable both modulation and evasion of the local immune response, sustaining persistent colonization.

Although BV is classically described as a clinically non-inflammatory condition, consistent evidence demonstrates that it is associated with a state of molecular inflammation, primarily characterized by increased levels of pro-inflammatory cytokines (IL-1β, IL-6, and TNF-α) and the pro-inflammatory CXC chemokine CXCL-8, a key mediator of neutrophil recruitment [16,22]. In the present study, IL-1β levels were significantly higher in intermediate microbiota and BV compared with normal microbiota, indicating that inflammatory activation associated with this cytokine begins in the transitional microbiota and persists in established dysbiosis.

Similar findings were reported by Santos-Greatti et al. [39], who also observed increased IL-1β levels in women with intermediate microbiota compared with those with *Lactobacillus*-dominated microbiota. These results suggest that intermediate microbiota represents a heterogeneous and transitional state, rather than merely an undefined profile, and highlight the importance of integrating quantitative bacterial measures with immunological parameters to better understand BV pathogenesis. Longitudinal studies have shown that alterations in cervical innate immune mechanisms may precede the establishment of vaginal dysbiosis, suggesting that the immune response actively participates in the microbial transition process [40]. This provides biological support for the elevated IL-1β levels observed in intermediate microbiota in our study, indicating that inflammatory activation may occur before clinically established BV and that immunological changes may accompany—or even precede—the microbial shifts observed during progression to BV.

When multivariate analyses were performed considering both the microscopic classification of the vaginal microbiota and the bacterial loads of the species evaluated, IL-1β levels remained associated with the presence of *F. vaginae*, independently of the microbiota profile. This finding suggests that, among the species assessed, *F. vaginae* has a closer relationship with the activation of the local IL-1β inflammatory response than *Gardnerella* spp., reinforcing its potential role as a modulator of vaginal inflammation during dysbiosis progression.

From a mechanistic perspective, experimental studies have demonstrated that *F. vaginae* is capable of inducing innate immune responses in vaginal epithelial cells, promoting the production of pro-inflammatory cytokines and antimicrobial peptides [41,42]. Moreover, this species shows a high capacity for integration into the BV-associated biofilm, contributing to its structural stability and persistence [21,43]. These factors help explain why the presence—and especially the increased abundance—of *F. vaginae* is associated with elevated IL-1β levels.

With respect to inflammatory chemokines, CXCL-8 showed significant variation among the vaginal microbiota patterns analyzed. Its levels were higher in intermediate microbiota compared with normal microbiota, while no statistically significant differences were observed between BV and normal microbiota or intermediate microbiota. This pattern suggests that increased CXCL-8 may be associated with a transient state of vaginal microbiota imbalance, characteristic of intermediate microbiota, rather than with fully established dysbiosis. The absence of CXCL-8 elevation in BV may reflect immunomodulatory mechanisms associated with biofilm organization and inflammatory evasion by bacteria predominant in this fully dysbiotic state. Given that CXCL-8 is a potent neutrophil chemoattractant and that BV is classically not associated with neutrophilic chemotaxis, unchanged levels of this chemokine in BV are expected.

In contrast, intermediate microbiota states may be associated with a distinct inflammatory profile characterized by greater activation of the innate immune response. Previous studies indicate that CXCL-8 production varies according to vaginal bacterial composition, rather than solely based on the clinical diagnosis of BV [44,45]. Moreover, different bacterial species have been shown to correlate differently with local CXCL-8 levels [44]. As CXCL-8 exhibits a negative correlation with *Lactobacillus* abundance [46], the increase observed in intermediate microbiota may reflect a partial reduction in these protective bacteria, without yet reaching the more immunologically silent profile typical of BV.

When CXCL-8 was analyzed using multivariate models that simultaneously considered vaginal microbiota diagnosis and bacterial loads, only the microbiota profile remained associated with its levels, while the presence of *Gardnerella* spp. or *F. vaginae* showed no independent association. This suggests that CXCL-8 production is linked to global characteristics of the microbial community rather than to these two individual taxa specifically. Furthermore, other conditions associated with bacterial compositions distinct from BV, such as aerobic vaginitis, have also been linked to increased CXCL-8 levels [45], reinforcing that this chemokine reflects an inflammation profile dependent on the type of microbiota present.

The other cytokines evaluated in this study did not differ among vaginal microbiota patterns, a result that may be related to the immunomodulatory behavior of biofilm formation. Evidence suggests that *G. vaginalis*, as a primary colonizer, can attenuate epithelial inflammatory responses during biofilm establishment [23], reducing activation of pathways such as CXCL-8, IL-6, and TNF-α. Studies using three-dimensional epithelial models also show that *G. vaginalis* alone does not robustly induce IL-6, CXCL-8, or TNF-α [47]. Nevertheless, other studies report broader inflammatory responses, including increased IL-1β, IL-6, CXCL-8, TNF-α, RANTES (Regulated on Activation, Normal T-cell Expressed and Secreted), and MIP-1β (Macrophage Inflammatory Protein-1β) in cell cultures exposed to *G. vaginalis* or *F. vaginae* [48,49]. Similarly, clinical studies, such as one conducted in South African women, described elevations in several cytokines (IL-1, IL-6, CXCL-8, TNF-α, and IL-10) in association with BV [50]. These discrepancies may reflect methodological differences, population variability, the influence of co-infections, intra-species diversity (e.g., *Gardnerella* species), or temporal differences between dysbiosis stages.

Finally, this study has some limitations that should be considered when interpreting the results. The sample size of intermediate microbiota cases was numerically smaller than that of normal microbiota and BV cases, which may have reduced the statistical power to detect differences among these profiles. In addition, *Gardnerella* quantification in this study was performed using F-GV1/R-GV3 primer set targeting a region spanning the 16S-23S rRNA intergenic spacer. Although this assay has been widely applied in clinical investigations, it was developed prior to the genome-based taxonomic revision of the genus *Gardenerella*. As ITS-based assays do not reliably discriminate among all currently recognized species within the genus, species-level identification cannot be fully assured. Therefore, the bacterial loads reported herein should be interpreted as representing *Gardnerella* spp. detected by this molecular target, rather than confirmed identification of a specific *Gardnerella* species under current taxonomy. Furthermore, bacterial analysis was restricted to selected taxa and did not include comprehensive characterization of vaginal microbial diversity, limiting the ability to assess the potential contribution of other microbial species to the observed inflammatory responses.

## 5. Conclusions

Taken together, the findings of this study indicate that progression to the dysbiotic state of BV does not depend solely on the presence of dysbiosis-associated microorganisms, but rather on the quantitative increase in key species—particularly *F. vaginae*—and their interaction with the vaginal inflammatory microenvironment. The early detection of *F. vaginae* in intermediate microbiota, together with elevated IL-1β levels, suggests that inflammatory alterations may begin before the establishment of full-blown BV, reflecting a transitional ecological and inflammatory state. These results reinforce the role of intermediate microbiota as a biologically active stage rather than merely an undefined profile and highlight the importance of quantitative and immunological approaches for understanding BV pathogenesis. From a clinical perspective, the identification of bacterial and inflammatory markers associated with dysbiosis progression may contribute to more accurate diagnostic strategies and to the development of targeted interventions aimed at preventing gynecological and obstetric complications associated with BV.

## Figures and Tables

**Table 1:** Sociodemographic and clinical characteristics of the study participants. Distribution of participants according to age, ethnicity, marital status, and symptoms, based on the microscopic classification of the vaginal microbiota (normal microbiota, intermediate microbiota, and bacterial vaginosis).

Variables	Normal Microbiota (n = 68)	Intermediate Microbiota (n = 24)	Bacterial Vaginosis (n = 60)
Age	37 (22–54)	39 (19–55)	41 (20–58)
Ethnicity *			
White	44 (63.2%)	12 (50.0%)	32 (53.3%)
Black	2 (2.9%)	1 (4.2%)	7 (11.7%)
Others	5 (7.4%)	4 (16.7%)	2 (3.3%)
Marital status **			
Single	25 (36.8%)	5 (20.8%)	23 (38.3%)
Married	25 (36.8%)	12 (50.0%)	18 (30.0%)
Vaginal discharge			
Yes	11 (16.2%)	9 (37.5%)	9 (15.0%)
No	40 (62.5%)	9 (37.5%)	37 (61.7%)
Vaginal odor			
Yes	4 (6.3%)	2 (8.3%)	6 (10.0%)
No	35 (51.5%)	13 (54.2%)	30 (50.0%)
Vaginal pruritus			
Yes	3 (4.4%)	2 (8.3%)	4 (6.7%)
No	40 (62.5%)	15 (62.5%)	37 (61.7%)

* Data available for 109 patients; ** Data available for 108 patients. Age values are presented as median (range).

## Data Availability

The original contributions presented in this study are included in the article. Further inquiries can be directed to the corresponding author.

## Abbreviations

The following abbreviations are used in this manuscript:

ATCC	American Type Culture Collection
BV	Bacterial vaginosis
CST	Community State Type
CT	Cycle threshold
CXCL-8	CXC chemokine ligand 8
HIV	Human Immunodeficiency Virus
HPV	Human Papillomavirus
IL	Interleukin
MIP-1β	Macrophage inflammatory protein 1β
RANTES	Regulated upon activation normal T expressed and secreted
TNF-α	Tumor Necrosis Factor α
